# Supplementary material for: Imaging Membrane Curvature inside a FcεRI-Centric Synapse in RBL-2H3 Cells Using TIRF Microscopy with Polarized Excitation
Source: J Imaging. Author manuscript; Available in PMC 2019 Jul 29. (PMC6663088; doi:10.3390/jimaging5070063)
Supplement: Supplementary file 1 [file NIHMS1041488-supplement-Supplemental.zip › supp mat/jimaging-439483-SuppMat.docx]

Supplementary Materials:

**Figure S1.** Image masks used to create normalized histograms of the number of pixels with particular P/S ratios in +IgE and –IgE regions shown in Figure 5d,h,l. Masks shown in the first row (panels a, b, and c) were used to create histogram shown in Figure 5d. Masks shown in the second row (panels d, e, and f) were used to create histogram shown in Figure 5h, and masks shown in the third row (panels g, h, and i) were used to create the histogram shown in Figure 5l. Masks shown in the first column represent cell masks obtained by thresholding the sum image of the P-polarized and S-polarized excitation. Masks shown in the second or middle column represent masks that contain IgE (+IgE) and were obtained by thresholding the IgE-488 image. Masks shown in the third column represent cell masks that lack IgE 
(-IgE) and were obtained by subtracting the corresponding +IgE masks from the cell masks in the first column.  Scale bar shown in panel a) applies to all nine panels and represents 2 µm.

**Figure S2.** Image showing composite of IgE signal (red) and P-S ratio (green) from a) Figure 5i and 5k and b) Figure 5a and 5c of the main text.
